# Editor's Letter-Box

**Published:** 1896-12-05

**Authors:** 


### EDITOR'S LETTER-BOX.

[Our Correspondents are reminded that prolixity is a great bar to publication, and that brevity of style and conciseness of statement greatly facilitate early insertion.]

MR. OSWALD J. MILNE writes: I wish to make you a suggestion with a view to encourage the giving of fresh cut flowers to the London hospitals, by facilitating their collection and distribution. There are a great many city gentlemen having gardens and greenhouses, living some distance out of town, who, especially at the rose, dahlia, and chrysanthemum seasons, have large surplus quantities that they hardly know what to do with. It has occurred to me that if such railway companies having suburban lines as, say the London and South-Western, at Waterloo, would allow a stall to be placed in a central position on the station for say two hours, nine to eleven each morning, and occupied by a representative of the hospitals, to receive flowers, and if the arrangement were well known, many gentlemen would, I venture to think, carry up cut flowers to hand over there, knowing that they would be appreciatively received, and go direct to the wards of the London hospitals. Fruit and game might also find its way up through the same agency.

